# Supplementary material for: The European Stag Beetle (Lucanus cervus) Monitoring Network: International Citizen Science Cooperation Reveals Regional Differences in Phenology and Temperature Response
Source: Insects. 2021 Sep 10;12(9):813. doi: 10.3390/insects12090813 (PMC8466357; doi:10.3390/insects12090813)
Supplement: Supplementary file 1 [file insects-12-00813-s001.zip › File S1.pdf]

Protocol for the European Stag Beetle Survey

Transect

Select a place where the beetle can be easily observed and where you have at least a path or route that you can easily follow. The transects should be 500 m long. Also choose a starting point that you can easily retrieve each year. The direction of the transect should be kept constant every time the transect is walked. Preferably, walk more or less eastwards so the sun is going down behind you.

Sampling period

Weekly transects should be walked, when weather conditions are suitable, for eight weeks during June and July. Stag beetles are rarely active below 12 °C and become fully active from 18 °C onwards. Therefore, transect walks are preferably done on warm (>12 °C) evenings with little or no rain and no strong wind. If the temperature does not rise above 12 °C, during one or more weeks, then there is no need to walk the transect. If you are on holiday or there are other reasons why you cannot walk the transect for a certain period, it is advisable to find somebody to replace you. The transect should be walked at least six times each year. Additional transect walks can be carried out, especially around the short period of peak activity. This peak period might vary between locations and from year to year but is often at the end of June or beginning of July and marked by sultry (warm) evenings.

A transect walk

The transect is 500 m long and should be walked from start to end at a gentle pace taking 30 min to complete it. It might be useful to organise a trial session to calibrate your speed with the aid of reference points (83.3 m per 5 min). The survey should start 15 min before sunset (the time of sunset for the nearest city can be calculated at www.timeanddate.com/sun/) (accessed on 1 August 2021). The transect is walked by only one surveyor that makes the observations and records them.

Before starting the transect note the:

- Transect name
- Date of transect walk
- Name of surveyor
- Sunset time
- Starting time
- Wind speed (Beaufort scale): 0 = no wind (0 Beaufort), 1 = Wind felt on exposed skin, smoke, leaves and flags move (1–2 Beaufort), 2 = dust and loose paper raised, small branches move, flags and clothes flutter (3–4 Beaufort), 3 = trees rustle, small trees and large branches move, empty plastic bins tip over (5–6 Beaufort) and 4 = Whole trees in motion, flags taut, effort needed to walk or bike against the wind (7 or more Beaufort).
- Initial temperature
- Initial relative air humidity (if possible)

Observations

Note all stag beetles (*Lucanus cervus*) within a virtual box of approximately 10 m in front of the surveyor and 5 m to each side of the surveyor. For each observation, note the time, number, sex and activity. Use M (male), F (female) or U (unknown) for sex and DR (dead/remains), CO (copulating), NF (non flying), FL (flying) and U (unknown, changing during observation or not notated) for activity. In some regions other *Lucanus* species are known to be present and in that case observations might be noted as L. spec. if species is uncertain. So observations could for example be written down as:

22h03: 1F DR (1 female dead/remains)

22h17: 2M FL (2 male flying)

Please collect any remains that you find of dead stag beetles. These can be used for further identification by a specialist and/or for genetic or morphometric studies. Label them and If possible

store them separately in pure ethanol (without ether) or in paper tissue for genetic analyses. Send the remains at the end of the season to the responsible at your country (see our website). Please take into account that in most of the countries the European stag beetle is protected by law. This means you might need a permit to collect dead stag beetles.

Field sheet

A pdf file is available to fill in the observations in the field at our website. Afterwards, observations should be reported via our website.

Yearly habitat assessment

In order to explain the trends of the different transects, we ask you to yearly assess the habitat in an area of about 200 m surrounding your transect. Habitat assessment implies only four questions. If you find it difficult to fill in these questions, please contact the responsible at your country.

What is the main habitat (200 m surrounding the transect):

- Urban (including industrial, residential, transport and commercial areas)
- Park (public or private park or open residential areas with lots of full grown trees)
- Agricultural
- Broad-leaved forest (less than 25% coniferous)
- Coniferous forest (less than 25% broadleaves)
- Mixed forest (mixture of broadleaves and coniferous trees)
- Natural or semi-natural vegetation other than forest

Which part of the area is covered by crowns of broad-leaved trees (200 m surrounding the transect): To estimate this, look at an aerial photograph of your transect for example via Google maps.

- 0–25%
- 26–50%
- 51–75%
- 76–100%

How much dead wood is available (200 m surrounding the transect): Notify that dead wood includes old trees (>1m in diameter), dead broad-leaved trees, broadleaved tree stumps and artificial logs (railway sleepers, log piles and others) of >40 cm in diameter

- No or little (<1/ha)
- Moderate (1–5/ha)
- High (5–25/ha)
- Very high (>25/ha)

Has the habitat (suitable dead wood) over the last five years been decreasing or increasing:

- Habitat is decreasing
- Habitat is rather stable
- Habitat is increasing
